# Active penetration of *Trypanosoma cruzi* into host cells: historical considerations and current concepts

**DOI:** 10.3389/fimmu.2013.00002

**Published:** 2013-01-25

**Authors:** Wanderley de Souza, Tecia M. Ulisses de Carvalho

**Affiliations:** ^1^Laboratório de Ultraestrutura Celular Hertha Meyer, Instituto de Biofísica Carlos Chagas Filho, Universidade Federal do Rio de JaneiroRio de Janeiro, Brazil; ^2^Instituto Nacional de Metrologia, Qualidade e TecnologiaRio de Janeiro, Brazil

**Keywords:** *Trypanosoma cruzi*, endocytosis, active penetration, phagocytosis, Chagas disease

## Abstract

In the present short review, we analyze past experiments that addressed the interactions of intracellular pathogenic protozoa (*Trypanosoma cruzi*, *Toxoplasma gondii*, and *Plasmodium*) with host cells and the initial use of the term active penetration to indicate that a protozoan “crossed the host cell membrane, penetrating into the cytoplasm.” However, the subsequent use of transmission electron microscopy showed that, for all of the protozoans and cell types examined, endocytosis, classically defined as involving the formation of a membrane-bound vacuole, took place during the interaction process. As a consequence, the recently penetrated parasites are always within a vacuole, designated the parasitophorous vacuole (PV).

## INTRODUCTION AND EARLY STUDIES

Investigation of the interactions of *Trypanosoma cruzi* with host cells became possible after techniques to cultivate this protozoan in tissue culture were developed. The first approach was described by Kofoid et al. (1935), who showed that the protozoan could survive and multiply in cultures of heart cells from mouse and rat embryos. A subsequent study by Romaña and Meyer (1942) using chick embryo heart cell cultures described the behavior of *T. cruzi* in tissue cultures in detail. Theirs *in vitro* observations of the interaction process in both living and fixed cultures led the authors to describe two mechanisms of cell infection by the protozoan: active penetration and phagocytosis. In this classic paper, it was stated that “in general, the active penetration was more visible with metacyclic forms that, with their great motility, easily crossed the cell surface, penetrating into the protoplasm of fibroblasts and myocytes.” To the best of our knowledge, this is the first reference to the active penetration of parasitic protozoans into cells. Six years later, Meyer and Xavier de Oliveira (1948) published a paper and a small book confirming the initial observations and reported that the parasite “can touch the surface of the host cells without penetration. Occasionally, they adhere to the cell surface and suddenly penetrate into the cell.” These observations were more clearly demonstrated in a classic film by H. Meyer and A. Barasa. Part of that film which is kept in our laboratory (founded in 1940 by Hertha Meyer) is available at the site of the Brazilian Society of Protozoology (www.sbpz.org.br). The same basic idea initially proposed by Meyer and colleagues was presented in 1973 by Dvorak and Hyde (Dvorak and Hyde, 1973; Hyde and Dvorak, 1973) based on observations of the interaction of *T. cruzi *trypomastigotes of the Ernestina strain with secondary bovine embryo skeletal muscle cells (BESM) and HeLa cells obtained through phase contrast microscopy under controlled conditions. The process of cell invasion was described as an active penetration process in which mechanical activity of the protozoan is prevalent, and the parasites penetrate into the cell through the plasma membrane. According to these studies, the parasites enter cells posterior end first.

It is important to note, although it is not the focus of the present review, that the same basic idea of active penetration has also been applied to other protozoans, such as *Toxoplasma gondii* and *Plasmodium*, which were again analyzed first by Nery Guimarães and Meyer (1942) and Dvorak et al. (1975).

## SECOND PHASE

One of the authors of this review (WS) joined the Hertha Meyer’s laboratory in 1969 and began the first studies addressing the interaction of *T. cruzi* with chick embryo heart muscle cells and macrophages using electron microscopy. Jim Dvorak visited the laboratory several times between 1972 and 1980, and intense discussions about the concept of active penetration took place at that time. With enthusiasm, Jim defended the idea that *T. cruzi* and *Plasmodium* are able to generate a transient tunnel-like structure in the host cell plasma membrane that is sealed immediately after parasite internalization, and the parasite then establishes intimate contact with the host cell cytoplasm. Up to this point, electron microscopy had not been used to analyze the parasitic protozoan–host cell interaction process. However, a few images obtained in our laboratory showed that recently penetrated trypomastigotes were not in contact with the myofibers of heart muscle cells but were instead located within a membrane-bound vacuole. These observations were made approximately in 1972 when Nadia Nogueira was being trained to work with *T. cruzi* in Hertha Meyer’s lab. Subsequently, she moved to Rockefeller University in New York, where she started a project in Zanvil Cohn’s laboratory analyzing the interaction of *T. cruzi* with several different cell types, including mouse peritoneal macrophages, HeLa cells, L cells, and calf embryo fibroblasts. During a visit to Dr. Cohn’s lab, one of the authors of the present paper (WS) had the opportunity to discuss these electron microscopy images with these investigators, which were subsequently published in a classic paper in 1976 (Nogueira and Cohn, 1976). The images showed clearly that all internalized parasites were initially located in a vacuole, designated the parasitophorous vacuole (PV) following the suggestion of William Trager, who described a vacuole akin to that in Nogueira and Cohn’s images in erythrocytes infected by *Plasmodium* (Langreth and Trager, 1973). Similar observations were made in *T. gondii* by Jones and Hirsch (1972). Since that time, it has become clear that in all situations examined to date, *T. cruzi* and certain other intracellular protozoan always penetrate the host cell in a process that is better characterized as an endocytic process, involving the initial formation of aPV. This process takes place independent of the nature of the host cell. Even so-called non-professional phagocytic cells can be penetrated by *T. cruzi* and *T. gondii.* The term induced endocytosis or even induced phagocytosis has been used to refer to a process in which the parasite is able to stimulate endocytic activity in the future host cell.

According to Nogueira and Cohn in the case of *T. cruzi*, subsequent to penetration, the parasites leave the PV in a process they described as “escaping” and then enter into direct contact with the host cell cytoplasm. However, a few years later, it was shown (Carvalho and de Souza, 1989) that there is no escaping, but rather, fragmentation of the PV membrane occurs, most likely due to the activity of enzymes secreted by the parasite, as was subsequently shown by Andrews et al. (1990), associated with the complete disappearance of the PV. Taken together, these observations clearly indicate that an endocytic process is always involved during the process of *T. cruzi* internalization into a host cell. The same basic idea can be extended to other intracellular parasitic protozoa.

## A NEW CONCEPT OF “ACTIVE PENETRATION”

We had the opportunity to discuss the data described above as well as the results obtained by several groups in *Plasmodium* and *Toxoplasma* with Hertha Meyer, James Dvorak, Nadia Nogueira, and Zanvil Cohn, and we reached the conclusion that active penetration, as initially defined, does not exist during the process of parasitic protozoa interacting with host cells. A possible exception is observed during infection of host cells by microsporidians, which include a large number of species initially considered to be protozoans but that have more recently been considered to be fungi based on the presence of a large number of genes that, upon phylogenetic analysis, cluster Microsporida with Fungi (see review by Xu and Weiss, 2005). These organisms present a complex life cycle, and their spores contain a structure known as the polar tube. When one of these organisms attaches to the surface of a host cell, signals activate a process that leads to evagination of a structure known as the polar tube, which then penetrates into the host cell, establishing a type of bridge between the cytoplasm of the spore and the cytoplasm of the host cell. The sporoplasm, which may contain one or two nuclei, then flows through the connecting tube and is transferred to the host cell cytoplasm, where it will develop.

After some discussion, it was concluded that the term active penetration could be used to indicate the fact that the parasite plays an important role in the “induction of the host cell invasion process.” Indeed, since the first description of the process, it has been clear that the intense movement of the protozoan, especially due to the flagellar beating process, plays some role. This idea was further analyzed by Schenkman et al. (1991), who showed that maintenance of an active energetic metabolism is fundamental for *T. cruzi* to invade cells, as this process is prevented by treatment of the parasites with 2-deoxy-glucose, an inhibitor of glycolysis, as well as sodium azide, antimycin, and oligomycin, which interfere with the mitochondrial metabolism involved in the synthesis of ATP.

The term active penetration has also been employed from another perspective. Kipnis et al. (1979), for example, used it to describe the penetration of bloodstream trypomastigotes into macrophages in a process that was only partially inhibited by cytochalasin B. At present, we know that this compound does not inhibit all forms of endocytosis.

Therefore, we can conclude that the available data, especially those obtained through transmission electron microscopy of thin sections, clearly show that recently penetrated *T. cruzi* of both infective and non-infective forms are always located within a PV that interacts with the organelles of the endosomal–lysosomal system during its short existence. This phenomenon occurs in all the cell types examined to date independent of whether they are professional or non-professional phagocytic cells. The formation of the PV involves the induction of a calcium flux into the host cell via the action of a parasite-derived calcium agonist, which is generated through the action of a parasitic oligopeptidase (Caler et al., 1998), as well as the synaptotagmin VII pathway (Caler et al., 2001), the recruitment of lysosomes (Tardieux et al., 1992), and the participation of microtubules (Tyler et al., 2005) and actin filaments (Rosestolato et al., 2002). Recently, Fernandes et al. (2011) showed that *T cruzi* trypomastigotes mimic the process of wound repair with Ca^2+^-dependent exocytosis of lysosomes by delivering acid sphingomyelinase to the host plasma membrane, facilitating parasite entry into host cells. These aspects of the invasion process have been extensively reviewed in recent years (Hall, 1993; Burleigh and Andrews, 1995; Yoshida, 2006; Alves and Colli, 2007; de Souza et al., 2010; Caradonna and Burleigh, 2011; Butler and Tyler, 2012; Fernandes and Andrews, 2012; Romano et al., 2012).

## ENDOCYTOSIS IS A COMPLEX BIOLOGICAL PROCESS

Our present knowledge of the endocytic process shows that it is more complex than previously thought. Indeed, in addition to the classical phagocytic process, there are several ways a cell can ingest extracellular material of variable dimensions. These mechanisms can be either dependent on dynamin, such as the clathrin- and caveolin-mediated processes, or independent of dynamin, as occurs during processes including macropinocytosis, and lipid raft-mediated endocytosis. It is likely that other mechanisms will be described in view of the large number of groups attempting to better characterize the endocytic process. As described in another review in this volume (Barrias et al., submitted), *T. cruzi* may use all of these mechanisms to enter host cells. It is possible that the parasite selects the mechanism to be used based on factors such as the nature of the cell and the host cell surface ligand to which binds. Once the parasite binds to and is recognized by the host cell surface it triggers a process that is better described as an induced endocytosis.

## Conflict of Interest Statement

The authors declare that the research was conducted in the absence of any commercial or financial relationships that could be construed as a potential conflict of interest.

## References

[B1] Alves M. J. Colli W. (2007). *Trypanosoma cruzi*: adhesion to the host cell and intracellular survival. *IUBMB Life* 59 274–279 17505965 10.1080/15216540701200084

[B2] Andrews N. W. Abrams C. K. Slatin S. L. Griffiths G. (1990). A *T. cruzi*-secreted protein immunologically related to the complement component C9: evidence for membrane pore-forming activity at low pH. *Cell* 61 1277–1287 2194668 10.1016/0092-8674(90)90692-8

[B3] Burleigh B. A. Andrews N. W. (1995). The mechanisms of *Trypanosoma cruzi* invasion of mammalian cells. *Annu. Rev. Microbiol.* 49 175–209 8561458 10.1146/annurev.mi.49.100195.001135

[B4] Butler C. E. Tyler K. (2012). Membrane traffic and synaptic crosstalk during host cell entry by *Trypanosoma cruzi*. *Cell. Microbiol.* 14 1345–1353 22646288 10.1111/j.1462-5822.2012.01818.x PMC3428839

[B5] Caler E. V. Vaena de Avalos S. Haynes P. A. Andrews N. W. Burleigh B. A. (1998). Oligopeptidase B-dependent signaling mediates host cell invasion by *Trypanosoma cruzi*. *EMBO J*. 17 4975–4986 9724634 10.1093/emboj/17.17.4975 PMC1170826

[B6] Caler E. V. Chakrabarti S. Fowler K. T. Rao S. Andrews N. W. (2001). The Exocytosis-regulatory protein synaptotagmin VII mediates cell invasion by *Trypanosoma cruzi*. *J. Exp. Med.* 193 1097–1104 11342594 10.1084/jem.193.9.1097 PMC2193425

[B7] Caradonna K. L. Burleigh B. A. (2011). Mechanisms of host cell invasion by *Trypanosoma cruzi*. *Adv. Parasitol.* 76 33–61 21884886 10.1016/B978-0-12-385895-5.00002-5

[B8] Carvalho T. M de Souza W. (1989). Early events related with the behaviour of *Trypanosoma cruzi* within an endocytic vacuole in mouse peritoneal macrophages. *Cell Struct. Funct.* 14 383–392 2553278 10.1247/csf.14.383

[B9] de Souza W. de Carvalho T. M. Barrias E. S. (2010). Review on *Trypanosoma cruzi*: host cell interaction. *Int. J. Cell Biol.* 2010, pii: 295394 10.1155/2010/295394 PMC2926652 20811486

[B10] Dvorak J. A. Hyde T. P. (1973). *Trypanosoma cruzi*: interaction with vertebrate cells *in vitro*. I. Individual interactions and the cellular and subcellular levels. *Exp. Parasitol.* 34 268–283 4583055 10.1016/0014-4894(73)90087-8

[B11] Dvorak J. A. Miller L. H. Whitehouse W. C. Shiroishi T. (1975). Invasion of erythrocytes by malaria merozoites. *Science* 187 748–750 803712 10.1126/science.803712

[B12] Fernandes M. C. Cortez M. Flannery A. R. Tam C. Mortara R. A. Andrews N. W. (2011). *Trypanosoma cruzi *subverts the sphingomyelinase-mediated plasma membrane repair pathway for cell invasion. *J. Exp. Med.* 208 909–921 21536739 10.1084/jem.20102518 PMC3092353

[B13] Fernandes M. C. Andrews N. W. (2012). Host cell invasion by *Trypanosoma cruzi*: a unique strategy that promotes persistence. *FEMS Microbiol. Rev.* 36 734–747 22339763 10.1111/j.1574-6976.2012.00333.x PMC3319478

[B14] Jones T. C. Hirsch J. G. (1972). The interaction between *Toxoplasma gondii* and mammalian cells. II. The absence of lysosomal fusion with phagocytic vacuoles containing living parasites. *J. Exp. Med.* 136 1173–1194 4343243 10.1084/jem.136.5.1173 PMC2139290

[B15] Hall B. F. (1993). *Trypanosoma cruzi*: mechanisms for entry into host cells. *Semin. Cell Biol.* 4 323–333 8257784 10.1006/scel.1993.1039

[B16] Hyde T. P. Dvorak J. A. (1973). *Trypanosoma cruzi*: interaction with vertebrate cells *in vitro*. II. Quantitative analysis of the penetration phase. *Exp. Parasitol.* 34 284–294 4583056 10.1016/0014-4894(73)90088-x

[B17] Kofoid C. A. Wood F. C. McNeil E. (1935). The cycle of *Trypanosoma cruzi* in tissue cultures of embryonic heart muscle. *Univ. Calif. Publ. Zool.* 41 23–24

[B18] Kipnis T. L. Calich V. L. G da Silva W. D. (1979). Active entry of bloodstream forms of *Trypanosoma cruzi* into macrophages. *Parasitology* 78 89–98 105337 10.1017/s0031182000048617

[B19] Langreth S. G. Trager W. (1973). Fine structure of the malaria parasite *Plasmodium lophurae*. *J. Protozool.* 20 600–613 10.1111/j.1550-7408.1973.tb03584.x 4128534

[B20] Meyer H Xavier de Oliveira M. (1948). Cultivation of *Trypanosoma cruzi* in tissue culture: a four year study. *Parasitology* 39 91–94 18876881 10.1017/s0031182000083591

[B21] Nery Guimarães F. Meyer H. (1942). Cultivo de *Toxoplasma*, Nicole & Manceaux, 1909, em culturas de tecidos. *Rev. Bras. Biol.* 2 123–129

[B22] Nogueira N. Cohn Z. (1976). *Trypanosoma cruzi*: mechanism of entry and intracellular fate in mammalian cells. *J. Exp. Med.* 143 1402–1420 775012 10.1084/jem.143.6.1402 PMC2190227

[B23] Romaña C. Meyer H. (1942). Estudo do ciclo evolutivo do *Schizotrypanum cruzi* em cultura de tecidos de embrião de galinha. *Mem. Inst. Oswaldo Cruz* 37 19–27

[B24] Romano P. S. Cueto J. A. Cassassa A. F. Vanrell M. C. Gotlieb R. A. Colombo M. I. (2012). Molecular and cellular mechanisms involved in the *Trypanosoma cruzi* host cell interplay. *IUBMB Life* 64 387–396 22454195 10.1002/iub.1019 PMC3709976

[B25] Rosestolato C. T. Dutra Jda M. de Souza W de Carvalho T. M. (2002). Participation of host cell actin filaments during interaction of trypomastigote forms of *Trypanosoma cruzi* with host cells. *Cell Struct. Funct.* 27 91–98 12207050 10.1247/csf.27.91

[B26] Schenkman S. Robbins E. S. Nussenzweig V. (1991). Attachment of *Trypanosoma cruzi* to mammalian cells requires parasite energy, and invasion can be independent of the target cell cytoskeleton. *Infect. Immun.* 59 645–654 1987081 10.1128/iai.59.2.645-654.1991 PMC257806

[B27] Tardieux I. Webster P. Ravesloot J. Boron W. Lunn J. A. Heuser J. E. (1992). Lysosome recruitment and fusion are early events required for trypanosome invasion of mammalian cells. *Cell* 71 1117–1130 1473148 10.1016/s0092-8674(05)80061-3

[B28] Tyler K. M. Luxton G. W. G. Appewhite D. A. Murphy S. C. Engman D. (2005). Responsive microtubule dynamics promote cell invasion by *Trypanosoma cruzi*. *Cell. Microbiol.* 7 1579–1591 16207245 10.1111/j.1462-5822.2005.00576.x

[B29] Yoshida N. (2006). Molecular basis of mammalian cells invasion by *Trypanosoma cruzi*. *Ann. Acad. Bras. Cienc.* 78 87–111 10.1590/s0001-37652006000100010 16532210

[B30] Xu Y. Weiss L. M. (2005). The microsporidian polar tube: highly specialized invasion organelle. *Int. J. Parasitol.* 35 941–953 16005007 10.1016/j.ijpara.2005.04.003 PMC3109658

